# Assessment of a Passive Exoskeleton for Neck and Lower Back Support: A Task Study on Muscle Activity and User Perceived Exertion

**DOI:** 10.3390/s26041354

**Published:** 2026-02-20

**Authors:** Niromand Jasimi Zindashti, Negar Riahi, Linda Miller, Mahdi Tavakoli, Hossein Rouhani, Ali Golabchi

**Affiliations:** 1 Department of Mechanical Engineering, University of Alberta, Edmonton, AB T6G 1H9, Canada; jasimizi@ualberta.ca (N.J.Z.); riahi@ualberta.ca (N.R.); hrouhani@ualberta.ca (H.R.); 2 EWI Works International Inc., Edmonton, AB T6E 3N8, Canada; lmiller@ewiworks.com (L.M.); alireza1@ualberta.ca (A.G.); 3 Department of Electrical & Computer Engineering, University of Alberta, Edmonton, AB T6G 1H9, Canada; 4 Department of Biomedical Engineering, University of Alberta, Edmonton, AB T6G 1H9, Canada; 5 Glenrose Rehabilitation Hospital, Edmonton, AB T5G 0B7, Canada; 6 Department of Civil and Environmental Engineering, University of Alberta, Edmonton, AB T6G 1H9, Canada

**Keywords:** passive neck exoskeleton, neck disorder, EMG analysis, rate of perceived exertion, ergonomic intervention

## Abstract

Musculoskeletal disorders, particularly in the neck and back, are prevalent across various professions, stemming from prolonged static postures and awkward neck flexions. This study investigated the efficacy of a passive exoskeleton, designed to alleviate musculoskeletal neck and back strain, in a simulated neck flexion task. Ten participants performed tasks involving neck flexion at angles of 15°, 30°, 45°, and 60°, both with and without the exoskeleton. Additionally, the impact of using a headlight was evaluated at a 45° neck flexion angle. Wearable electromyography sensors were used to quantify muscle activity as an indicator of neuromuscular loading, while subjective discomfort was assessed using the Rate of Perceived Exertion scale, and endurance times were recorded. The results demonstrated significant reductions in neck and lower back muscle activity (median values up to 31.0%) and perceived discomfort (median values up to 50.0%), with the most improvements at 30° and 45° neck flexion angles. Participants reported 50% higher endurance time when using the exoskeleton. Minimal benefits were observed at 15° flexion, likely due to reduced musculoskeletal demand at this angle. These findings highlight the potential of exoskeletons as an ergonomic intervention to mitigate neck and back strain in occupations where high degrees of neck flexion are prevalent.

## 1. Introduction

Musculoskeletal disorders are a major occupational health concern, affecting millions of workers worldwide and leading to substantial economic burdens due to lost productivity, medical expenses, and compensation claims [1]. Among these disorders, neck musculoskeletal disorders are prevalent occupational health issues across various occupations, which often involve prolonged static postures with sustained head flexion. For example, the nature of surgery, requiring precision, stability, and long durations of concentration, places significant strain on the spine and neck, increasing the risk of chronic pain, discomfort, and long-term health complications. According to a survey of general surgeons, 83% of surgeons suffer from neck-related discomfort [2]. The results from [3,4,5] also show that musculoskeletal disorders are correlated with surgeons’ absence from work, and most of them have concerns regarding early retirement. Analyzing over 110 surgical procedures, one study reported that surgeons frequently adopt risky neck postures in over 40% of laparoscopic and 80% of open surgeries [6]. Quantitative analyses of surgeons further revealed high neck flexion angles (27–33°), levels associated with an increased risk of neck musculoskeletal disorders [7]. To mitigate these risks, various interventions have been explored, including ergonomic workstation modifications and scheduled breaks.

While these interventions offer some benefits, they also have notable limitations. For example, ergonomic adjustments [8,9] cannot provide support for different postures. Moreover, even with optimal ergonomic setups, individuals may still experience fatigue and strain from prolonged or repetitive tasks. Scheduled breaks, often recommended to reduce fatigue [10,11,12,13], can provide temporary relief, but their effectiveness depends on compliance and how individuals recover. Moreover, breaks may not be feasible as they can affect the individuals’ accuracy and interrupt their work process. Given these limitations, there is a need for alternative solutions that provide continuous, real-time support without requiring major workflow disruptions.

Exoskeletons, wearable devices designed to provide external support, hold potential as an alternative intervention for reducing neck strain, potentially replacing previously explored options such as work breaks and arm supports. Exoskeletons have already demonstrated notable benefits across various occupations and body regions [14,15], particularly in tasks that place significant stress on the lower back [16]. For instance, studies show that back support exoskeletons can reduce the physical load on the back by up to 60% [17,18], contributing to decreased musculoskeletal strain and improved comfort. These promising results suggest that similar devices could be adapted to address the ergonomic challenges faced due to neck flexion.

While exoskeletons have been well explored in many industrial sectors, fewer studies [19,20] have explored the efficacy of neck support exoskeletons for healthcare occupations (e.g., surgery) where professionals are exposed to a heightened risk of neck-related musculoskeletal disorders. Moreover, while the majority of surgeons rely on headlights during procedures, few studies have yet evaluated how exoskeletons interact with this additional device to replicate realistic operating conditions.

In surgical environments, neck strain often presents a primary concern for surgeons. However, lower back strain also poses significant risks due to extended periods of standing and leaning forward. To address these concerns, exoskeletons could be designed to provide targeted support for both the neck and lower back, effectively distributing the load and reducing musculoskeletal stress on these critical areas. While different exoskeletons with different designs have been developed [20,21], not all of them are designed to support both the neck and lower back. The recently introduced NekSpine™ exoskeleton claims to address neck strain while offering additional lower back support. This exoskeleton is primarily designed to alleviate the load on neck muscles. Yet it also targets and provides support for the lower back by (1) preventing excessive forward curvature bending, which helps maintain proper posture, (2) transferring part of the lower back load to the hips, reducing the strain on the lower back. Thus, the exoskeleton may reduce musculoskeletal stress in the neck and lower back parts of the body.

A recent study has evaluated the effectiveness of this exoskeleton. The results show that the exoskeleton can reduce discomfort and postural risks [22]. While promising, the results mainly rely on subjective evaluations, and the exoskeleton is evaluated in dynamic tasks. However, the results from another study show that the effectiveness of exoskeletons depends on the postural angles [20]; this is important as surgeons maintain long static postures at different neck flexion angles. Additionally, the effectiveness of lower back support provided by the exoskeleton was not examined.

To complement the current literature, this study investigated the effectiveness of the NekSpine exoskeleton in reducing neck and lower back muscle activity at various neck flexion angles, collected by electromyography (EMG) sensors, and discomfort reported by users, during a simulated surgical task. In addition, the influence of headlight use together with the exoskeleton was examined by evaluating muscle activity and comparing scenarios with and without the exoskeleton and headlight. Furthermore, endurance time was measured to complement physiological and perceptual outcomes and to provide insight into sustained task performance. This approach aimed to provide a comprehensive understanding of the exoskeleton’s impact under conditions that closely replicate real surgical environments.

The key contributions of this study are as follows:

- This study provides an objective and subjective assessment of an exoskeleton’s ability to reduce muscle activity and discomfort in both the neck and lower back.
- The exoskeleton was evaluated at multiple flexion angles.
- The study evaluated the effects of using a headlight.

## 2. Materials and Methods

### 2.1. Participants

Ten participants (five males and five females, age: 26 ± 3 yrs, body height: 172 ± 8 cm, and body mass: 68 ± 10 kg) were recruited for data collection. All participants were able-bodied adults, and none of them had previous back or neck disorders. All signed a consent form after the experiment was explained to them. This study was approved by the research ethics board of the University of Alberta, ID: Pro00109264.

### 2.2. Exoskeleton

**Figure 1:**
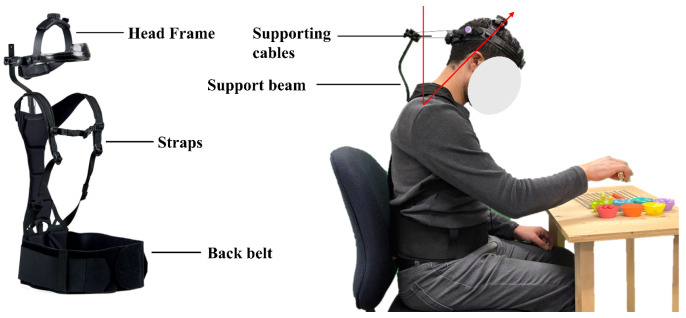
The exoskeleton used in this study for supporting the neck and lower back during head flexion postures.

The exoskeleton used in this study was NekSpine™ (San Clemente, CA, USA), which supports both the neck through the head frame and the lower back through the support beam along the spine. The total weight of the exoskeleton is less than 0.9 kg. It allows head rotations as the cable holding the head can slide within the connector to the support beam. Additionally, the amount of support for each head flexion angle can be set by continuously adjusting the pre-tension of the cables. The support beam comes in two sizes for different anthropometrics. The fitting of the head frame can also be carried out with two screws (Figure 1).

### 2.3. Experimental Procedure

Before the experiment, participants performed some trials with the exoskeleton to become familiar with its setting and the amount of support it provides. EMG sensors (Tringo Avanti EMG sensor, Delsys, Natick, MA, USA) were attached bilaterally to different muscle groups: the neck extensors, including the Splenius Capitis (SC) and Trapezius (T), as well as the lower back muscle, Latissimus Dorsi. These muscles are selected as they are the main contributors of neck and lower back flexion.

Before collecting data, participants underwent Maximum Voluntary Contraction (MVC) tests to determine the maximum achievable EMG amplitude. A dynamic procedure, as described in [23], was followed to assess lower back muscle MVC. For neck muscles (SC and T), participants performed the MVC test by pushing their head backward against a resisted force. To collect data, the participants were asked to hold their heads in flexion angles of 15°, 30°, 45°, and 60° for five minutes (Figure 1). These neck flexion angles were selected to represent a range of postures spanning low to high ergonomic risk levels commonly observed in surgical occupations [7]. To ensure accurate neck angles, a goniometer was used, with 0° defined as the natural resting position of the neck. Participants were then instructed to flex their heads forward from this position until they reached the specified angle. The task was performed with and without the exoskeleton at four neck flexion angles, resulting in eight task conditions. Also, two more conditions were explored: with and without the exoskeleton while wearing glasses equipped with a headlight (0.17 kg) at a head flexion angle of 45°.

In total, the participants performed the task under 10 conditions in a randomized order. Breaks of 5 min were considered between trials to prevent fatigue effects. For the condition of using the exoskeleton, participants were asked to set the support provided by the exoskeleton based on their own preferences at each head flexion angle. During the five-minute trials, participants simulated a task by sorting small colored balls using different grippers (Figure 1).

Along with EMG data, with a frequency of 2148 Hz, participants’ feedback was collected using a Rate of Perceived Exertion (RPE) scale (1–10) for both neck and lower back areas. After each trial, participants verbally reported their RPE scores to the experimenter. At the end of the experiment, participants underwent an endurance test at a head flexion angle of 45°. They were asked to stay at a head flexion angle of 45° with and without the exoskeleton until they felt fatigued. Participants recovered substantially (minimum 10 min) between the two endurance tests.

### 2.4. Data Analysis

First, all EMG data were processed using a 4th-order Butterworth band-pass filter, with cutoff frequencies set at 10 Hz and 500 Hz, to remove noise and preserve the signal of interest. EMG amplitudes were then normalized (nEMG) using the corresponding MVC values. The mean nEMG values were averaged across both sides of the body because the activity was symmetrical with respect to the body’s sagittal plane. EMG data, participants’ RPE feedback, and endurance time were used to compare the various aspects of the exoskeleton, comparing data with and without the exoskeleton ($\frac{Exo-NoExo}{NoExo}\times 100\%$).

A Shapiro–Wilk test was used to check the data normality. As the data were not normally distributed, non-parametric tests were used for all statistical analyses. Wilcoxon’s signed-rank and Wilcoxon’s rank-sum tests were used to compare the medians of paired and independent samples, respectively. The significance level was set at α = 0.05.

## 3. Results

This section presents the objective results derived from muscle activity measured through EMG sensors as well as the subjective outcomes based on RPE feedback and endurance times. Furthermore, a comparative analysis of the results between male and female participants is included to explore potential differences.

### 3.1. Muscle Activity from EMG Sensors

**Figure 2:**
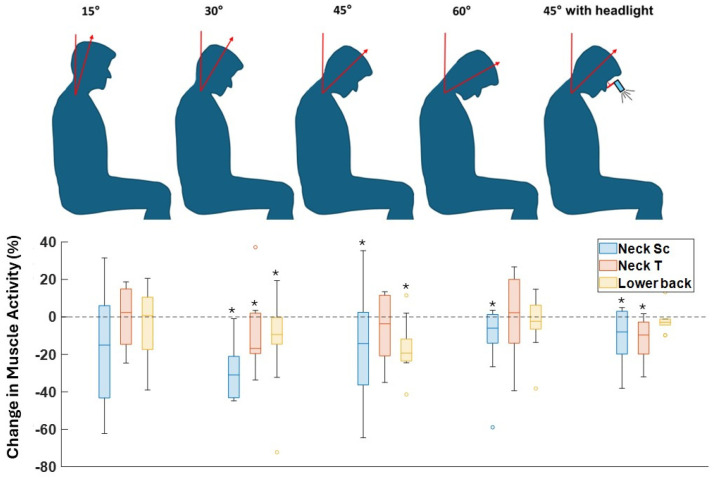
Change in neck and lower back muscle activity in different postures with and without the exoskeleton (SC: Splenius Capitis, T: Trapezius). * means a significant difference between results with and without the exoskeleton from the Wilcoxon signed-rank test (α = 0.05).

As Figure 2 highlights, reductions in neck and lower back muscle activity were observed when using the exoskeleton compared to conditions when it was not used. The median of lower back muscle activity decreased from 0% to 19.3% as the neck flexion angle grew from 15° to 45° (Table 1). However, this trend changes at a neck flexion angle of 60°, where the median of lower back muscle activity decreased by only 2.4%. In contrast, the largest reduction in neck muscle activity had a median of 31.0%, occurring at a neck flexion angle of 30°. Muscle activity did not show a significant decrease at the neck flexion angle of 15° for all three muscle groups. Furthermore, the exoskeleton effectively alleviated the load on both lower back and neck muscles even when the users wore the headlight. Specifically, it achieved significant reductions of 8.0% and 9.7% in the median of neck muscle activity (SC and T) and of 2.8% in the median reduction in lower back muscle activity.

### 3.2. Rate of Perceived Exertion (RPE)

**Figure 3:**
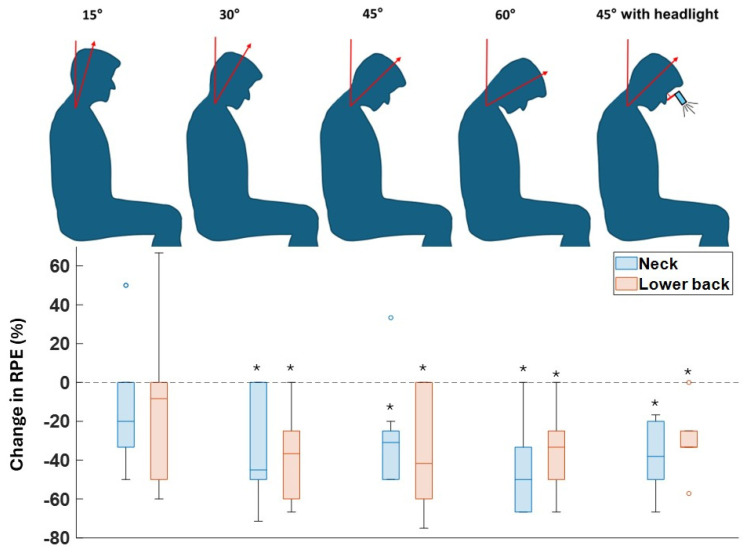
Changes in the RPE scale reported by users in the neck and lower back areas of the body in different task conditions with and without the exoskeleton. * means a significant difference between results with and without the exoskeleton from the Wilcoxon signed-rank test (α = 0.05).

As shown in Figure 3 and Table 1, participants reported decreased perceived discomfort and exertion in both the neck and lower back when using the exoskeleton. Notably, the median reduction in neck RPE increased from 20.0% at 15° neck flexion to 50.0% at 60° flexion. For the lower back, it increased from 8.3% to 41.7% as the neck flexion angle grew from 15° to 45°. However, the median reduction in lower back RPE in neck flexion angle of 60° (33.3%) was lower than that of 45° (41.7%). Furthermore, the participants experienced less discomfort while using the exoskeleton with a headlight, reporting median reductions of 38.1% for the neck and 33.3% for the lower back (Figure 3). RPE reductions while wearing the exoskeleton were significant in both body parts and all task conditions, except for a neck flexion angle of 15°.

### 3.3. Endurance Time

**Figure 4:**
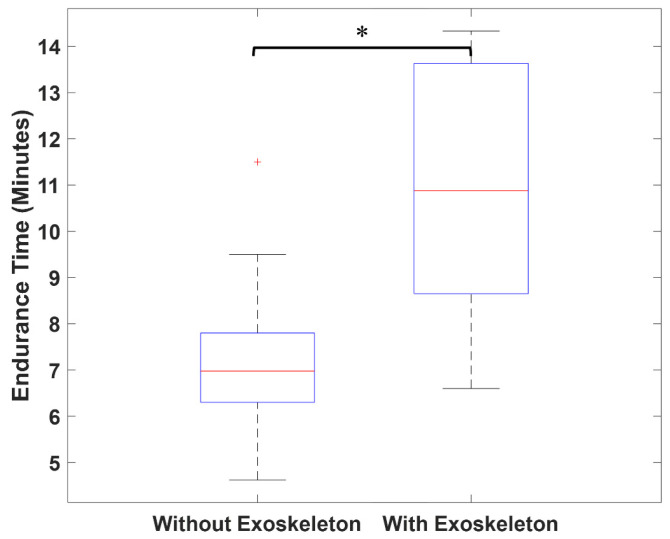
Endurance time reported by participants with and without the exoskeleton at a head flexion angle of 45°. * means a significant difference between results with and without the exoskeleton from the Wilcoxon signed-rank test (α = 0.05).

As shown in Figure 4, the participants demonstrated significantly longer endurance times when using the exoskeleton (*p* < 0.05). Specifically, the median endurance time was 7.0 min without the exoskeleton and 10.9 min with the exoskeleton.

### 3.4. Females vs. Males

**Figure 5:**
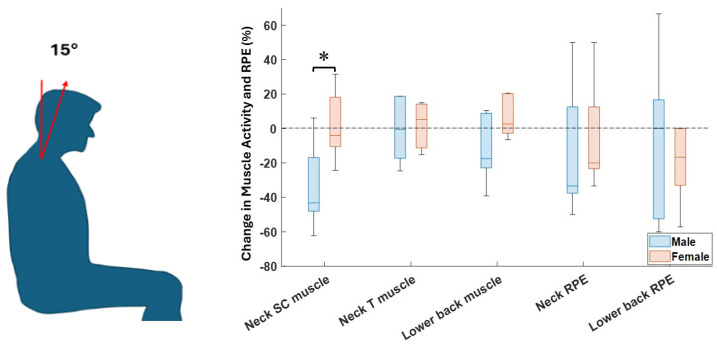

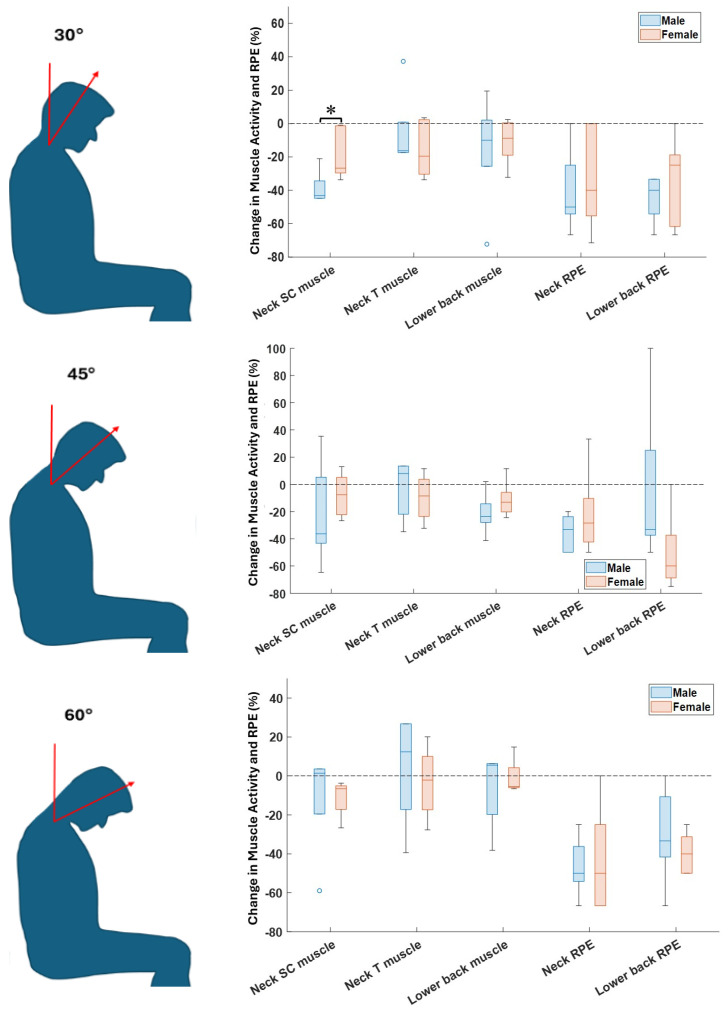

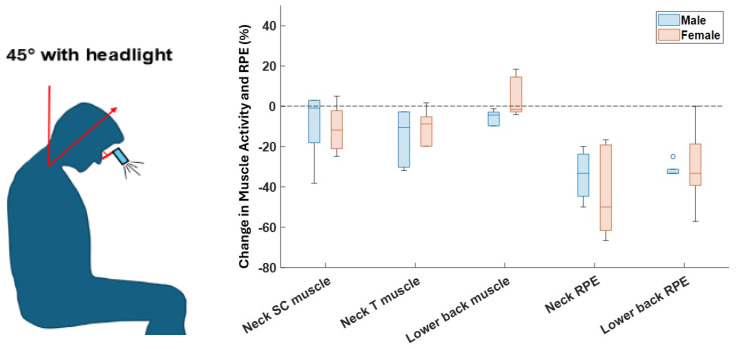
Changes in muscle activity and RPE feedback in male and female groups when using the exoskeleton (SC: Splenius Capitis, T: Trapezius). * means a significant difference between the results of male and female participants from the Wilcoxon rank-sum test (α = 0.05).

As shown in Figure 5, there were no significant differences between males and females when comparing changes in different objective and subjective variables and task conditions, except for the neck SC muscle at neck flexion angles of 15° and 30°.

## 4. Discussion

While exoskeletons have been widely adopted for various occupational tasks, there is limited research on their application in tasks involving sustained neck flexion. Professionals such as surgeons, dentists, and construction workers often adopt awkward neck flexion postures and experience significant discomfort in the neck and lower back, highlighting a critical need for ergonomic solutions. This study evaluated an exoskeleton, designed to reduce neck and, to a lesser extent, lower back strain during tasks involving neck flexion at varying angles. Both subjective feedback (the RPE scale and endurance time) and objective measurements (data from EMG sensors) were collected to comprehensively assess the exoskeleton’s performance.

First, data from EMG sensors highlighted the efficacy of the exoskeleton, showing up to a 37.3% reduction in neck extensor muscle activity (Figure 2), aligning with previous studies [20]. Interestingly, while lower back muscle activity reduction by using the exoskeleton increased with greater neck flexion angles, the neck muscle activity demonstrated a different pattern. The maximum median reduction in neck muscle activity occurred at a neck flexion angle of 30°, indicating a peak in the exoskeleton’s effectiveness for neck support at this specific posture. At smaller flexion angles (e.g., 15°), the head’s gravitational load is minimal, making exoskeleton support less essential, while at larger angles (e.g., 60°), increased muscle activation might be required to maintain head stability.

Second, the exoskeleton was effective in reducing neck muscle activity when it was worn along with glasses equipped with a headlight (Figure 2). While wearing a headlight posed an additional load on the neck and lower back muscles, the exoskeleton was effective in canceling out not only the moment due to the head weight but also the moment due to the headlight weight. Users reported up to 66.6% less discomfort when using the exoskeleton in the conditions of having the headlight (Figure 3).

Furthermore, recognizing users as a critical factor in the adoption of exoskeletons [19], participants generally found the device helpful in alleviating load and discomfort in the lower back and neck (Figure 2 and Figure 3). However, for tasks involving 15° of neck flexion, the users did not perceive a reduction in neck discomfort by using the exoskeleton. One possible explanation is that 15° of neck flexion does not impose a significant load on the neck, making the exoskeleton feel more like an additional weight than an assistive device in this condition. The results are supported by previous studies on the impact of exoskeletons on shoulder, neck, and lower back discomfort, where exoskeletons have decreased the level of discomfort in simulated surgery tasks [20].

Additionally, in most cases, the changes in objective and subjective variables differed, with changes in median RPE reduction being greater than those of muscle activity. While part of this discrepancy may stem from the inherent subjectivity of RPE, it underscores that objective measures alone do not fully capture users’ perceptions. Similarly, the results from [24] show that there is no significant correlation between changes in muscle activity and comfort or discomfort. Additionally, the results from [25] found that the mapping between muscle activity and RPE is not one-to-one and subjective evaluations capture aspects of perceived effort and fatigue that objective activity does not consistently reflect. This highlights the importance of users’ preferences in exoskeleton design and effectiveness assessment, as users are the central parts of exoskeleton adoption.

Although a 60° neck flexion angle represents the most extreme position compared to 15°, 30°, and 45°, the muscle activity results show smaller reductions at this angle compared to other flexion angles, particularly 30° and 45°. This discrepancy can be attributed to the way the exoskeleton provides support. Achieving higher neck flexion angles requires users to actively push against the exoskeleton to reach the desired position. Despite participants being trained and instructed to adjust the exoskeleton settings for both comfort and optimal support during neck flexion, maintaining this balance becomes increasingly challenging at higher flexion angles. Consequently, slight deviations in the exoskeleton’s settings can impact neck stability at high flexion angles. To maintain a stable head posture, both the extensor and flexor muscles are engaged, resulting in smaller reductions in muscle activity during a 60° neck flexion. Additionally, this limited support can be attributed to the flexion–relaxation phenomenon, in which the neck extensor muscles reduce EMG activity at near-maximum neck flexion [26]. This phenomenon was found to be occurring at a 60° neck flexion angle in [27].

Participants reported a 1.55-times-higher median endurance time when using the exoskeleton compared to not using it at a neck flexion angle of 45° (Figure 4). Since surgeons often maintain static postures for extended periods, the observed increase in endurance time highlights the potential of the exoskeleton to provide meaningful support during prolonged tasks, thereby reducing physical strain and improving overall comfort. A similar study evaluating a head support exoskeleton found that using the exoskeleton significantly increases the fatigue threshold time [28].

Finally, as demonstrated in in Figure 5, a significant difference between males and females was observed only at two neck flexion angles and exclusively for the SC muscle group. This limited difference suggests that, for most flexion angles and muscle groups, factors such as muscle adaptation, body posture, and exoskeleton support influence both males and females in a comparable manner. While the limited sample size for the two groups reduces the statistical power to detect any true differences, previous studies have shown that sex differences in neck muscle activity vary with posture and are not consistent across all conditions, highlighting task-specific differences in neck muscle behavior [29,30]. Additionally, regarding the evaluation of exoskeletons, the results from [31] showed that the exoskeleton’s effectiveness can differ between males and females because of variations in strength, endurance, and movement patterns. Females may benefit more from exoskeletons that support precision and fine motor control tasks, whereas males may experience greater advantages from exoskeletons that enhance strength and load-bearing capabilities.

Beyond its ergonomic role, the neck support exoskeleton can be conceptualized as a partial gravitational unloading intervention. Unloading paradigms have been widely employed in gravitational physiology as Earth-based analogs of microgravity (e.g., head down tilt, dry immersion, and bed rest) [32,33], where reductions in axial loading are known to alter muscle tone, proprioceptive input, and neuromuscular control strategies. Such unloading has been associated with reduced extensor muscle activity, shifts in flexor–extensor balance, and a tendency toward forward-flexed neutral postures observed in spaceflight conditions. From this perspective, the reduction in cervical extensor demand observed with the exoskeleton may not solely reflect ergonomic optimization but may also represent a neuromuscular adaptation to partial unloading. While decreased muscular demand may be beneficial for fatigue mitigation, prolonged unloading could influence sensorimotor control and postural regulation through mechanisms analogous to functional deafferentation. Therefore, the interpretation of head–neck kinematic changes should consider both ergonomic and gravitational modulation effects. Future research should investigate the long-term neuromechanical consequences of sustained unloading, particularly in precision-demanding occupational tasks such as surgical practice.

It should be noted that the exoskeleton used in this study is a passive device, and therefore the magnitude of mechanical support provided to the head was not directly quantified in the present study. The assistance level depends on cable pre-tension and head flexion angle, resulting in variable and task-dependent unloading of the head. Future studies should incorporate direct force measurements to quantify the actual extension moment generated by the exoskeleton. Such quantification would enable the more precise evaluation of load-sharing between the head and the exoskeleton.

This study demonstrated the effectiveness of an exoskeleton, designed mainly for neck support and partially for lower back support, in a simulated surgical task. However, its findings have limitations that can be addressed by further research. One limitation of this study is the sample size. Future studies should involve a larger, more diverse participant pool to draw more robust, generalizable conclusions. Second, a broader range of objective and subjective measures should be used to comprehensively evaluate the efficacy of exoskeletons across all relevant aspects. Future research should incorporate additional variables, such as energy expenditure and posture-based metrics, particularly since previous research suggests that body kinematics can be influenced by fatigue over time [34]. This approach will ensure a more thorough understanding of exoskeleton performance. Third, a surgical task was simulated for five minutes in this study. In reality, surgeons are exposed to different neck flexions in various body postures for much longer times, often while maintaining high levels of concentration, fine motor control, and visual attention. These cognitive demands may influence their perception of fatigue differently than in this study. Therefore, expanding upon this research, future studies can explore the performance and usability of exoskeletons with real surgeons while also considering the potential impact of mental workload on fatigue perception.

## 5. Conclusions

The exoskeleton used in this study demonstrated potential as an ergonomic intervention for reducing neck and lower back musculoskeletal strain during tasks involving moderate to high neck flexion angles. The results showed that the exoskeleton effectively decreased muscle activity, as measured by EMG sensors, and perceived discomfort in the neck and lower back, a critical occupational challenge faced by industry and healthcare professionals, such as surgeons and dentists. The exoskeleton also improved endurance, enabling users to maintain static postures for extended periods, which is essential for surgical tasks. Overall, the results show that neck support exoskeletons can offer a promising pathway to improve comfort, reduce strain, and potentially enhance long-term occupational well-being across various occupations involving neck flexion tasks.

## Figures and Tables

**Table 1:** Changes (%) in muscle activity and RPE scale (median [25% 75%] interquartile) when using the exoskeleton.

			Task Condition				
	Body Area		15°	30°	45°	60°	Headlight
Muscle Activity	Neck	SC	−15.1 [−43.2 6.0]	−31.0 [−43.1 −21.0]	−14.2 [−36.3 2.3]	−6.0 [−14.0 1.3]	−8.0 [−19.9 3.0]
		T	2.4 [−24.7 15]	−16.9 [−19.5 1.9]	−3.7 [−20.8 11.5]	2.2 [−14.0 20.0]	−9.6 [−19.8 −2.7]
	Back		0.6 [−17.4 10.5]	−9.4 [−14.6 −0.3]	−19.3 [−23.6 −11.8]	−2.3 [−6.5 6.3]	−2.8 [−4.4 −1.2]
RPE Scale	Neck		−20.0 [−33.3 0.0]	−45.0 [−50.0 0.0]	−31.0 [−50.0 −25.0]	−50.0 [−66.7 −33.3]	−38.1 [−50.0 −20]
	Back		−8.3 [−50.0 0.0]	−36.7 [−60.0 −25.0]	−41.7 [−60.0 0.0]	−33.3 [−50.0 −25.0]	−33.3 [−33.3 −25]

## Data Availability

The datasets presented in this article are available upon request.

## Abbreviations

The following abbreviations are used in this manuscript:

EMG	Electromyography
Exo	Exoskeleton
MVC	Maximum Voluntary Contraction
nEMG	Normalized muscle activity
NoExo	Without exoskeleton
RPE	Rate of Perceived Exertion
SC	Splenius Capitis (muscle)
T	Trapezius

## References

[1] Bevan S. (2015). Economic Impact of Musculoskeletal Disorders (MSDs) on Work in Europe. Best Pract. Res. Clin. Rheumatol..

[2] Szeto G.P.Y., Ho P., Ting A.C.W., Poon J.T.C., Cheng S.W.K., Tsang R.C.C. (2009). Work-Related Musculoskeletal Symptoms in Surgeons. J. Occup. Rehabil..

[3] Davila V.J., Meltzer A.J., Hallbeck M.S., Stone W.M., Money S.R. (2019). Physical Discomfort, Professional Satisfaction, and Burnout in Vascular Surgeons. J. Vasc. Surg..

[4] Howarth A.L., Hallbeck M.S., Lemaine V., Singh D.J., Noland S.S. (2019). Work-Related Musculoskeletal Discomfort and Injury in Craniofacial and Maxillofacial Surgeons. J. Craniofacial Surg..

[5] Wells A.C., Kjellman M., Harper S.J.F., Forsman M., Hallbeck M.S. (2019). Operating Hurts: A Study of EAES Surgeons. Surg. Endosc..

[6] Yang L., Money S.R., Morrow M.M., Lowndes B.R., Weidner T.K., Fortune E., Davila V.J., Meltzer A.J., Stone W.M., Hallbeck M.S. (2020). Impact of Procedure Type, Case Duration, and Adjunctive Equipment on Surgeon Intraoperative Musculoskeletal Discomfort. J. Am. Coll. Surg..

[7] Hallbeck M.S., Law K.E., Lowndes B.R., Linden A.R., Morrow M., Blocker R.C., Cain S.M., Degnim A.C., Hieken T.J., Jakub J.W. (2020). Workload Differentiates Breast Surgical Procedures: NSM Associated with Higher Workload Demand than SSM. Ann. Surg. Oncol..

[8] Galleano R., Carter F., Brown S., Frank T., Cuschieri A. (2006). Can Armrests Improve Comfort and Task Performance in Laparoscopic Surgery?. Ann. Surg..

[9] Steinhilber B., Hoffmann S., Karlovic K., Pfeffer S., Maier T., Hallasheh O., Kruck S., Seibt R., Rieger M.A., Heidingsfeld M. (2015). Development of an Arm Support System to Improve Ergonomics in Laparoscopic Surgery: Study Design and Provisional Results. Surg. Endosc..

[10] Zindashti N.J., Martinez K.B., Golabchi A., Tavakoli M., Rouhani H. (2024). A Predictive Equation for Maximum Acceptable Efforts Based on Duty Cycle in Repetitive Back-Involved Tasks. J. Saf. Res..

[11] Beltran Martinez K., Nazarahari M., Rouhani H. (2023). Breaking the Fatigue Cycle: Investigating the Effect of Work-Rest Schedules on Muscle Fatigue in Material Handling Jobs. Sensors.

[12] Engelmann C., Schneider M., Kirschbaum C., Grote G., Dingemann J., Schoof S., Ure B.M. (2011). Effects of Intraoperative Breaks on Mental and Somatic Operator Fatigue: A Randomized Clinical Trial. Surg. Endosc..

[13] Vijendren A., Devereux G., Tietjen A., Duffield K., Van Rompaey V., Van de Heyning P., Yung M. (2020). The Ipswich Microbreak Technique to Alleviate Neck and Shoulder Discomfort during Microscopic Procedures. Appl. Ergon..

[14] Alemi M.M., Madinei S., Kim S., Srinivasan D., Nussbaum M.A. (2020). Effects of Two Passive Back-Support Exoskeletons on Muscle Activity, Energy Expenditure, and Subjective Assessments During Repetitive Lifting. Hum. Factors.

[15] Golabchi A., Riahi N., Fix M., Miller L., Rouhani H., Tavakoli M. (2023). A Framework for Evaluation and Adoption of Industrial Exoskeletons. Appl. Ergon..

[16] Golabchi A., Jasimi Zindashti N., Miller L., Rouhani H., Tavakoli M. (2023). Performance and Effectiveness of a Passive Back-Support Exoskeleton in Manual Material Handling Tasks in the Construction Industry. Constr. Robot..

[17] Kuber P.M., Abdollahi M., Alemi M.M., Rashedi E. (2022). A Systematic Review on Evaluation Strategies for Field Assessment of Upper-Body Industrial Exoskeletons: Current Practices and Future Trends. Ann. Biomed. Eng..

[18] Golabchi A., Chao A., Tavakoli M. (2022). A Systematic Review of Industrial Exoskeletons for Injury Prevention: Efficacy Evaluation Metrics, Target Tasks, and Supported Body Postures. Sensors.

[19] Cha J.S., Monfared S., Stefanidis D., Nussbaum M.A., Yu D. (2020). Supporting Surgical Teams: Identifying Needs and Barriers for Exoskeleton Implementation in the Operating Room. Hum. Factors.

[20] Tetteh E., Hallbeck M.S., Mirka G.A. (2022). Effects of Passive Exoskeleton Support on EMG Measures of the Neck, Shoulder and Trunk Muscles While Holding Simulated Surgical Postures and Performing a Simulated Surgical Procedure. Appl. Ergon..

[21] Garosi E., Mazloumi A., Jafari A.H., Keihani A., Shamsipour M., Kordi R., Kazemi Z. (2022). Design and Ergonomic Assessment of a Passive Head/Neck Supporting Exoskeleton for Overhead Work Use. Appl. Ergon..

[22] Kim J.Y., Norasi H., Cassivi S.D., Black D.M., Hallbeck M.S. (2025). Use of an Intraoperative Head, Neck, and Back Support Exoskeleton on Surgeons’ Pain and Posture. Ann. Surg..

[23] Wang X., Beltran Martinez K., Golabchi A., Tavakoli M., Rouhani H. (2023). A Dynamic Procedure to Detect Maximum Voluntary Contractions in Low Back. Sensors.

[24] Kuijt-Evers L.F.M., Bosch T., Huysmans M.A., De Looze M.P., Vink P. (2007). Association between Objective and Subjective Measurements of Comfort and Discomfort in Hand Tools. Appl. Ergon..

[25] Rewitz K., Schindler S., Wolff W. (2024). Examining the Alignment between Subjective Effort and Objective Force Production. PLoS ONE.

[26] Maroufi N., Ahmadi A., Mousavi Khatir S.R. (2013). A Comparative Investigation of Flexion Relaxation Phenomenon in Healthy and Chronic Neck Pain Subjects. Eur. Spine J..

[27] Nimbarte A.D., Zreiqat M.M., Chowdhury S.K. (2014). Cervical Flexion–Relaxation Response to Neck Muscle Fatigue in Males and Females. J. Electromyogr. Kinesiol..

[28] Garosi E., Kazemi Z., Mazloumi A., Keihani A. (2024). Changes in Neck and Shoulder Muscles Fatigue Threshold When Using a Passive Head/Neck Supporting Exoskeleton During Repetitive Overhead Tasks. Hum. Factors.

[29] Reddy C., Zhou Y., Wan B., Zhang X. (2021). Sex and Posture Dependence of Neck Muscle Size-Strength Relationships. J. Biomech..

[30] Chen Y.-L., Chan Y.-C., Alexander H. (2024). Gender Differences in Neck Muscle Activity during Near-Maximum Forward Head Flexion While Using Smartphones with Varied Postures. Sci. Rep..

[31] Wollesen B., Gräf J., De Bock S., Alfio E., Díaz M.A., De Pauw K. (2024). Gender Differences in Performing an Overhead Drilling Task Using an Exoskeleton—A Cross-Sectional Study. Biomimetics.

[32] Watenpaugh D.E. (2016). Analogs of Microgravity: Head-down Tilt and Water Immersion. J. Appl. Physiol..

[33] Navasiolava N.M., Custaud M.-A., Tomilovskaya E.S., Larina I.M., Mano T., Gauquelin-Koch G., Gharib C., Kozlovskaya I.B. (2011). Long-Term Dry Immersion: Review and Prospects. Eur. J. Appl. Physiol..

[34] Bonakdar A., Houshmand S., Martinez K.B., Golabchi A., Tavakoli M., Rouhani H. (2025). Fatigue Assessment in Multi-Activity Manual Handling Tasks through Joint Angle Monitoring with Wearable Sensors. Biomed. Signal Process. Control.

